# How Does Water Availability Affect the Allocation to Bark in a Mediterranean Conifer?

**DOI:** 10.3389/fpls.2019.00607

**Published:** 2019-05-21

**Authors:** Ruth C. Martín-Sanz, Roberto San-Martín, Hendrik Poorter, Antonio Vázquez, José Climent

**Affiliations:** ^1^Sustainable Forest Management Research Institute (UVa-INIA), Palencia, Spain; ^2^Escuela Técnica Superior de Ingenierías Agrarias, Universidad de Valladolid, Palencia, Spain; ^3^Plant Sciences (IBG-2), Forschungszentrum Jülich GmbH, Jülich, Germany; ^4^Department of Biological Sciences, Macquarie University, North Ryde, NSW, Australia; ^5^Department of Forest Ecology and Genetics, Forest Research Centre (INIA-CIFOR), Madrid, Spain

**Keywords:** allocation, allometry, bark thickness, fire adaptation, fire ecology, genotype-environment interaction, phenotypic plasticity, Pinaceae

## Abstract

Bark thickness is a key structural feature in woody plants in the protection against fire. We used 19 provenances of *Pinus halepensis*, an obligate-seeder species, in a replicated common garden at two environments contrasting in water availability to assess the interacting effects of site environment and population in the relative allocation to bark, expecting lower allocation at the drier site. Secondly, given the average fire frequency, we analyzed whether trees reached the critical absolute thickness soon enough for population persistence via aerial seed bank. Our analyses indicated that trees at the moister site allocated a rather fixed quantity of resources independent of tree size, and almost all populations reached critical absolute bark thickness to eventually survive fire. In contrast, at the drier site allocation to bark reduced with tree size, and most populations did not reach the critical bark thickness. Populations from areas with higher fire frequency had thicker basal bark, while those from areas with severe droughts and short vegetative periods, had thinner bark. In conclusion, drought-stressed trees have a higher risk to die from fires before achieving reproduction and building a sufficient aerial seed bank.

## Introduction

Bark is a key structural feature in woody plants, and the correlation between bark thickness and fire regime has recently attracted increasing interest by plant scientists (Pausas, 2015, 2017; Rosell, 2016). Bark includes all tissues outside the vascular cambium and is formed by two main components with contrasting functions: the living inner bark, which is related to transport and storage of photosynthates, and the dead outer bark which protects the tree from fires, pathogens and herbivores, reduces water loss or provides structural support (for details see, Evert and Eichhorn, 2006; Graves et al., 2014; Romero, 2014; Schafer et al., 2015; Rosell, 2016). However, despite its functional relevance, the role of bark in plant ecological strategies and the causes of its variation remain poorly understood.

Bark thickness is the most studied bark property so far, due to its correlation with cambial insulation and protection, especially against fires (van Mantgem and Schwartz, 2003; Bauer et al., 2010; Hoffmann et al., 2012; Lawes et al., 2013; Pausas, 2015). The degree of heat insulation by bark increases with the square of its thickness (Hare, 1965; Vines, 1968; Peterson and Ryan, 1986). Therefore, bark thickness strongly decreases the thermal diffusivity –the most important bark protective property (Vines, 1968).

How costly bark is compared to other plant parts (especially, wood) is still poorly known. While bark and wood densities can be very similar in some species (like in conifer trees, Miles and Smith, 2009), the two tissues have different physical properties and chemical composition. Putatively high costs imply that the relative resource allocation to bark must be subjected to strong evolutionary trade-offs between the fitness benefits of a thicker bark and its costs at different ontogenetic stages (Schwilk and Ackerly, 2001).

Several studies have addressed the evolutionary consequences of bark thickness in tropical ecosystems, comparing from a few to many species in natural populations and focusing on species whose strategy against fire is adult survival (Richardson et al., 1990; Lawes et al., 2011a, 2013; Dantas and Pausas, 2013; Poorter et al., 2014; Rosell et al., 2014; Rosell, 2016). So far, few studies have addressed the intraspecific variation in bark thickness (Climent et al., 2004; Tapias et al., 2004; Stephens and Libby, 2006; Briand et al., 2014), and even fewer have used common garden experiments where genetic and environmental effects can be properly separated (Matziris, 2000; Tapias et al., 2004; Stephens and Libby, 2006; Kohnle et al., 2012). As far as we know, bark variation has only been related to different fire regimes, mainly surface fires (Keeley and Zedler, 1998; Jackson et al., 1999; Schwilk et al., 2013; Graves et al., 2014; Pausas, 2015), whereas the environmental factors determining bark thickness and allometry have barely been investigated (but see Jager et al., 2015; Richardson et al., 2015). Determining the possible phenotypic plasticity of bark thickness separately from genetic differences within species is fundamental to improve our understanding of the trade-offs related to bark that can limit adaptive evolution under a changing climate.

Resource allocation in plants changes along ontogenetic trajectories; therefore, distinguishing between environmental effects from purely developmental differences is critical when studying plasticity in allocation (Poorter and Nagel, 2000; Wright and McConnaughay, 2002; Weiner, 2004). While accounting for ontogenetic changes in such long-lived plants like trees is elusive, the concepts and theory of allometry are probably the best available tools. There are different methods to study plant allometric patterns (Poorter and Sack, 2012), but most are based on logarithmically-transformed power law allometric equations (Niklas, 1994; Ter-Mikaelian and Korzukhin, 1997). This procedure has been followed in some studies of bark allometry and allocation to bark, normally using the residuals of the log-log bark thickness-tree diameter regression (see Paine et al., 2010; Rosell, 2016). Alternative approaches have also been applied, such as the relative bark thickness (2 × absolute bark thickness divided by tree diameter; Midgley and Lawes, 2016; see Pausas, 2015, Supplementary Information for details on different methods).

Despite the relevance of differential allocation to bark during a plant’s development, the survival of a plant facing a given fire depends on its *absolute* bark thickness rather than on the *relative* one (Midgley et al., 2010; Lawes et al., 2011b). The critical absolute bark thickness above which a tree can survive different types of fire is a highly useful parameter (VanderWeide and Hartnett, 2011; Wesolowski et al., 2014; Dehane et al., 2015; Pausas, 2015; Madrigal et al., 2019). As mentioned, above, thermal diffusivity of bark is strongly determined by its thickness (see Hare, 1965; Vines, 1968), but is independent of bark structure, density or moisture content (Martin, 1963; Uhl and Kauffman, 1990; Pinard and Huffman, 1997). In forest trees, bark thickness is generally studied at breast height (see, Harmon, 1984; Stephens and Libby, 2006; Rosell et al., 2015 for exceptions). However, bark thickness at the trunk base is ecologically relevant since trees can die by the girdling of their basal stem part in surface fires, even of low intensities (Jones et al., 2004).

We focused our research on *Pinus halepensis* Mill., a well-studied model species in Mediterranean fire-prone ecosystems (Ne’eman et al., 2004). This species is an obligate-seeder, which lacks resprouting ability. Its vital strategy is based on early building of an aerial seed bank, rather than on adult tree survival (Tapias et al., 2001; Ne’eman et al., 2004; Pausas and Keeley, 2014). However, this species is mentioned in the literature both as thin- and as moderately thick-barked (Fernandes et al., 2008; Chambel et al., 2013; Grivet et al., 2013) and some works report variable survival to low or moderate intensity fires (Ducrey et al., 1996; Trabaud and Valina, 1998; Rigolot, 2004; Fernandes et al., 2008) which coincides with our own field observations. Absolute bark thickness was observed to be moderately heritable in this species (Matziris, 2000), which implies the existence of genetic variability, but differentiation among populations and phenotypic plasticity in this trait is still unknown. Moreover, considering the contrasting natural fire regimes between eastern and western Europe (natural fires are frequent in western Europe, while virtually all fires in eastern Europe are anthropogenic, Ne’eman et al., 2004), we expected intraspecific variation for bark thickness in *P. halepensis*.

The life expectancy of *P. halepensis* trees is usually between 20 and 50 years (Agee, 1998; Vázquez de la Cueva and Moreno, 1998; Arianoutsou, 2001; Tessler, 2012), but it can be as low as 6 years under short fire return intervals (Tessler, 2012). Moreover, *P. halepensis* needs between 15 and 20 years to achieve a sufficient aerial bank of mature seeds (Moreira et al., 2011) to ensure population persistence in case of fire. Therefore, the age of the trees sampled in the present work –18 years– is key given the life-history of the species. The fact that, at this age, this species might be capable of surviving fires of low or moderate intensity thanks to a thick enough bark at the base or at breast height is of paramount importance to avoid immaturity risk. We took advantage of an adult *P. halepensis* provenance trial replicated in two sites with high contrast of water availability due to a combination of climate and soil characteristics. We used 19 range-wide, natural populations of the studied species, which allowed us to disentangle genetic and environmental effects. We wanted to know whether environmental limitations could hamper reaching a thick enough bark to survive fires until accumulating a sufficient aerial seed bank that ensures population persistence. Thus, our first objective is to check the existence of different allocation to bark among populations under two contrasting test environments, conducive to different absolute bark thickness for each population and site both at the tree base and at breast-height. Our second objective is to determine whether provenance bark traits are related to the climate conditions and/or to fire history at provenances’ origin, indicative of local adaptation.

## Materials and Methods

### Research Sites

**FIGURE 1:**
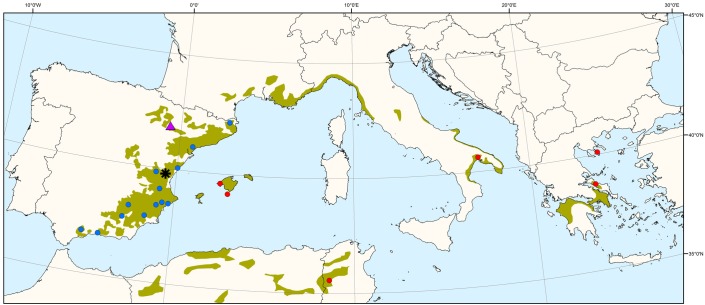
Distribution map of *Pinus halepensis* source populations (blue circles: 13 Iberian populations; red circles: 6 non-Iberian populations) and common garden experiments (purple triangle: drier site; black asterisk: moister site). The green area indicates the species’ natural distribution range.

A common garden experiment was set up in 1997 using a strictly replicated experimental design at different sites in eastern and central Spain. The trial includes 52 *P. halepensis* populations from continental Spain, Balearic Islands (Spain), France, Italy, Greece and Tunisia, thus covering most of the species’ range (see Climent et al., 2008 for details). We chose a subset of 19 populations (Figure 1 and Supplementary Table S1) representing the main geographic and environmental gradients of origin, and with a good balance among them. Previous studies showed major significant differences among *P. halepensis* populations, with eastern populations displaying faster growth and lower investment in early reproduction (including low serotiny) and populations toward the southwest with the opposite trends (Climent et al., 2008; Santos-del-Blanco et al., 2013). One-year-seedlings were finally planted in a row-column design in four randomized complete blocks with four plants per population and block (16 trees per population). Height, diameter and cone number were periodically measured, enabling absolute and relative growth assessments and reproductive allocation estimations.

**FIGURE 2:**
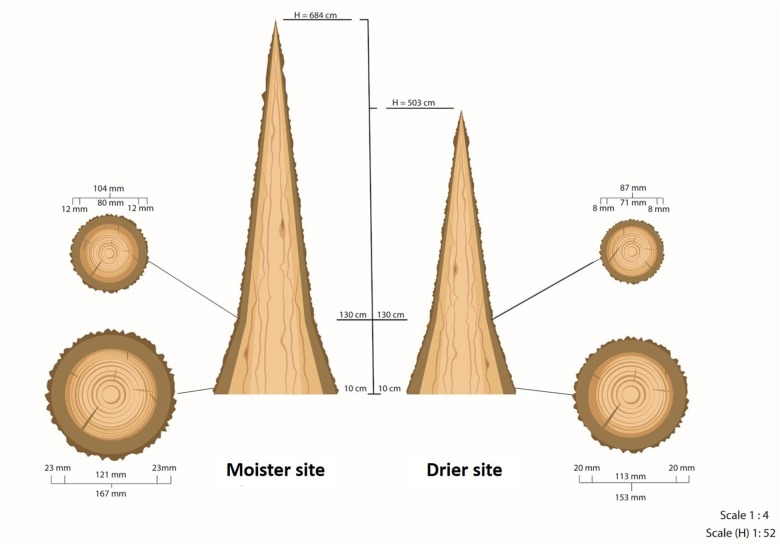
Scheme of the average tree at each of the test sites. H is the total height of the tree. Diameters and bark thicknesses of the circular cross-sections were measured at 10 and at 130 cm from the ground. This scheme does not represent the real taper of wood and bark along the trunk above 130 cm due to the lack of measurements at higher heights. This taper is neither constant nor homogeneous.

We selected two sites with contrasting environments allowing to separate population differentiation from phenotypic plasticity of bark thickness. Both sites differ in a range of environmental variables (Table 1), with drier site showing a 20% lower precipitation, a 100% higher drought intensity index (K) and more frequent and intense wind. Both sites contrast in soil structure, depth and water retention, which leads to a strong divergence in water availability. By contrast, soil nutrient availability between sites is very similar (Gil et al., 1996). Therefore, we refer to these differences as “drier” and “moister.” Both potential forest productivity (Sánchez-Palomares and Sánchez-Serrano, 2000) and survival were higher at the moister site (1.8-fold in productivity and 9% more survival compared to the drier site; Martín-Sanz et al., 2016; Figure 2 and Table 2). Moreover, site also affected significantly reproduction, degree of serotiny (long-lasting closed cones in the crown) and tree form.

**Table 1:** Main climatic characteristics of the two test sites of the *Pinus halepensis* common garden experiment (*n* = 512 trees).

Site	Latitude	Longitude	Altitude (m)	P (mm)	T (°C)	MTWM (°C)	MTCM (°C)	A (months)	K	WS (m/s)	WSS (m/s)	Soil type	PFP (m3/ha/year)	DLI (mol/m^2∗^day)	Sp_DLI (mol/m^2∗^day)	A_DLI (mol/m^2∗^day)
Drier	41° 52′ 22″ N	0° 38′ 54″ W	423	402	14.1	24.1	5.5	3.14	0.45	5.60	4.90	Gypsic Xerosol	2.25–3.00	31.10	36.0	23.7
Moister	39° 49′ 29″ N	01 34′ 22″ W	605	509	14.4	22.9	7.8	2.51	0.22	4.93	3.94	Calcic Cambisol	4.50–5.25	31.15	35.2	25.2

*P, annual rainfall; T, mean annual temperature; MTWM, mean temperature of the warmest month; MTCM, mean temperature of the coldest month; A, drought duration parameter; K, drought intensity parameter, calculated as the ratio between the dry area (when 2T > P) and the wet area of the climograph (when 2T < P); all previous variables from Gonzalo-Jiménez (2010); WS, mean annual wind speed at 80 m above surface; WSS, mean summer wind speed at 80 m above surface (from Aymamí et al., 2011); Soil type classification according to FAO guidelines (FAO, 2015); PFP, Potential Forest Productivity, calculated following the Paterson Climate Index and obtained from the Potential Forest Productivity Map of Spain (Sánchez-Palomares and Sánchez-Serrano, 2000); DLI, Daily Light Integral; Sp_DLI and A_DLI, Daily Light Integral for the vegetative period (spring and autumn, respectively). DLI data from New et al. (1999).*

**Table 2:** Mean values and standard errors (SE) of different growth variables at both test sites.

	Moister (*n* = 271)	*SE*	Drier (*n* = 241)	*SE*
H (cm)	684.0	7.9	503.7	5.0
D10 (mm)	167.1	2.3	153.3	1.7
D130 (mm)	103.9	2.0	87.2	1.3
BT10 (mm)	23.5	0.3	20.7	0.2
BT130 (mm)	12.4	0.3	8.0	0.2

*H, total tree height; D10, diameter at tree base (at 10 cm); D130, diameter at breast height (at 130 cm); BT10, bark thickness at tree base; BT130, bark thickness at breast height. All variables were significantly different between sites: P < 0.0001.*

### Sampling and Variable Description

Sampling was carried out in both experimental sites when trees were 18 years old (271 and 241 trees were sampled at the “moister” and “drier” sites, respectively). Stem diameter and bark thickness were measured basally (10 cm above the ground; hereafter referred as D10 and BT10, respectively) and at breast height (hereafter D130 and BT130, respectively) using a tree caliper and a standard bark gauge (Supplementary Figure S1). Since we were obliged to preserve the common garden experiment we abstained from more precise, but invasive or destructive bark measuring methods like extraction of bark portions (Jackson et al., 1999; Paine et al., 2010; Graves et al., 2014) or cutting stem discs to remove the bark (Rosell et al., 2014, 2015; Rosell, 2016). Therefore, by bark thickness (basal: BT10 or at breast height: BT130) we refer to total (inner and outer) bark. We performed a preliminary sampling of a few trees at each test site measuring bark thickness at three points surrounding the bole. We found that measuring bark thickness at a single point was accurate enough given the uniformity of *P. halepensis* bark at this age. Thus, for each height, we measured bark at a single point of the bole, always in the south orientation.

While simple and straightforward, we think that the linear relationship between bark thickness and diameter does not represent accurately enough the real resource allocation to bark. Our point is that allometry of bark allocation should be rather assessed through variables that are as-close-as-possible to the biomass of bark and sapwood (the functional part of the trunk). Therefore, focusing purely on the allometry of bark allocation, we looked at the allometric relationship between bark and sapwood volumes, basing the analysis on the relationship of percentage of bark volume and total volume from tree base to breast height (Martín-Sanz, 2018). Our sampled trees did not contain any heartwood (see **Appendix A** and Supplementary Figure A.1), so total volume is bark and sapwood (functional xylem) volumes.

Firstly, we calculated the total volume for the cone trunk from the tree base to breast height with the equation for a circular truncated cone (in dm^3^):

(1) $${\text{V}}_{\text{T}}=\pi /3*\text{h}*({\text{Rb}}^{2}+\text{Rb}*\text{Rbh}+{\text{Rbh}}^{2})$$

where h is the height difference between the base (10 cm above ground) and breast height (130 cm), Rb is the basal radius and Rbh is the radius at breast height.

Sapwood volume (dm^3^) was computed with the following equation:

(2) $${\text{V}}_{\text{S}}=\pi /3*\text{h}*({\text{SRb}}^{2}+\text{SRb}*\text{SRbh}+{\text{SRbh}}^{2})$$

where h is the height difference between the base (10 cm above ground) and breast height (130 cm), SRb is the sapwood basal radius and SRbh is the sapwood radius at breast height.

Finally, bark volume (dm^3^) and percentage of bark volume were estimated by Equations (3) and (4), respectively:

(3) $${\text{V}}_{\text{B}}={\text{V}}_{\text{T}}-{\text{V}}_{\text{S}}$$

(4) $${\text{\%V}}_{\text{B}}={\text{V}}_{\text{B}}/{\text{V}}_{\text{T}}*100$$

### Statistical Analysis

General linear mixed models (LMM) were fit with PROC MIXED procedure in SAS software (SAS Institute Inc., 2015). The incorporation of variables into the models was checked using the Akaike’s Information Criterion (AIC), selecting those models with lower AIC value. This criterion favors model fit and simplicity, based on the principle of parsimony (fewer parameters in the model). Residuals of each model were examined for normality using diagnostic plots and Kolmogorov-Smirnov normality tests.

### Allometric, Plastic, and Genetic Effects on Bark Allocation

We compared means of percentage of cone trunk from tree base to breast height taken up by bark volume (Equation 4) by site and populations using a LMM with environmental effect represented by trial sites and genetic effect by populations. In addition, we did the same model adding total volume of the cone trunk as a covariate in order to look for tendencies in percentage of bark volume. In both models, block within site was used as random factor.

Additionally, we wanted to compare our approach with the most commonly applied allometric method, thus we also used model II regression, i.e., standardized major axis (SMA) to examine the allometry of bark volume vs. the total volume of the cone trunk from the tree base to breast height (Henry and Aarssen, 1999; Niklas, 2006; Warton et al., 2006).

The classical allometric model is V_B_ = aV_T_^b^ and is usually fit as logV_B_ = log_10_a + b log_10_V_T_ (Huxley, 1932; Niklas, 1994; Ter-Mikaelian and Korzukhin, 1997). Parameter *a* is the elevation or allometric coefficient and parameter *b* is the regression slope or allometric exponent. An exponent significantly different from 1 indicates an allometric relationship between the variables studied (increasing or decreasing with size, non-constant). The allometric exponents of each population at each site were compared with the isometric coefficient (*b* = 1) and to one another by multiple *post hoc* comparisons. These analyses were carried out with the smatr package (Warton et al., 2012) implemented in R software v3.3.2. (R Core Team, 2016). As the SMA analysis indicated that slopes differed among populations and sites, a general SMA test of elevation (allometric parameter) differences was not necessary.

### Plastic and Genetic Effects on Bark Thickness at Breast and Basal Height

We analyzed the effects of population (genetic effect), experimental site (environment) and their interaction on absolute bark thickness at breast height and at tree base with LMM, including block within site as random factor. We compared the population per site variation of absolute bark thickness with critical thickness values –the thickness above which the risk of cambium damage decreases considerably, therefore indicating a threshold for tree survival– both at breast height and at tree base. While our bark thickness data did not differentiate between inner and outer bark neither we compared further heat insulating properties as moisture content or density, we assumed that bark anatomy should not differ significantly among provenances of a single species, as found in other species (see Wesolowski et al., 2014). Due to the lack of experimental data of critical bark thickness for *P. halepensis*, we used data published for the genetically closest available tree species: a high thickness of 20 mm, postulated to allow survival to moderate fires (for *Pinus pinea*; Madrigal et al., 2019) and a low thickness of 10 mm that would allow survival only to low-intensity fires (van Mantgem and Schwartz, 2003; VanderWeide and Hartnett, 2011).

### Relationship of Bark Thickness With Seed Source Environment

Looking for ecotypic trends in bark thickness, we tested for correlations among the mean values for bark thickness of our raw data (at breast and basal heights) and continuous environmental variables, as well as with fire frequency records from the 13 Iberian populations. The nineteen original bioclimatic variables for populations’ origin were obtained from Worldclim v.1.4 (Hijmans et al., 2005). We considered nine of these variables: annual precipitation, summer precipitation (June, July, and August), spring precipitation (March, April, and May), autumn precipitation (September, October, and November), precipitation of the driest month, annual mean temperature, mean temperature of the warmest month, mean temperature of the coldest month, and a continentality index (difference between mean temperature of the warmest month and mean temperature of the coldest month). Three spatial variables were also recorded for each population: longitude, latitude and altitude. We carried out a principal components analysis (PCA) with *varimax* rotation to reduce the number of environmental variables and allow a more synthetic interpretation. We decided the number of principal components to retain running a parallel analysis with 1000 iterations (Hayton et al., 2004) and selecting those principal components with eigenvalues for observed data larger than those from simulations. The analysis was performed with psych package (Revelle, 2017) on the R software v3.3.2. (R Core Team, 2016). Selected variables with loadings above 0.80 were used in Spearman correlation analysis with bark thickness. Natural fire frequency data (lightning fires, of relevance in the east part of Spain; Vázquez de la Cueva and Moreno, 1998) were defined as the number of fires in 90,000 ha of surface covered by *P. halepensis* forests each year during the period 1974–2010. A 10 × 10 km grid-unit was used to derive this fire frequency data for the 13 Iberian populations of *P. halepensis.* Unfortunately, we lacked data sources of, similarly, reliable and thorough fire information for the non-Iberian populations. Spearman correlations were done with Hmisc package (Harrell et al., 2018), implemented in R software v3.3.2. (R Core Team, 2016).

## Results

### Allometric, Plastic, and Genetic Effects on Bark Allocation

**FIGURE 3:**
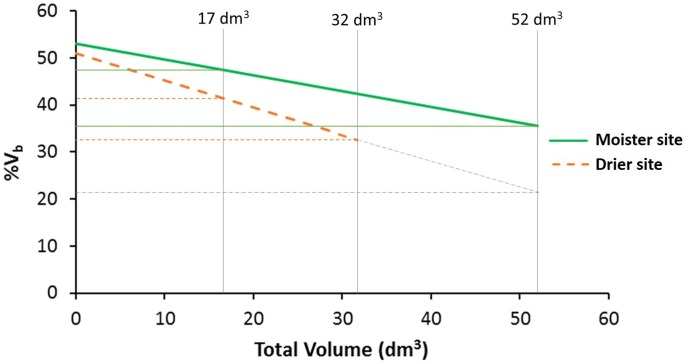
Percentage of bark volume vs. total volume per site. Vertical lines indicate the global mean total volume for both sites (17 dm^3^), and the maximum total volume reach at each test site (32 dm^3^ at the drier site and 52 dm^3^ at the moister site). Gray dot-dash lines represent the percentage of bark volume in the drier site if the trees would reach the same maximum total volume as in the moister site. At higher total volume, the difference between sites increases (*n* = 512 trees).

Trees at the drier site showed a lower percentage of bark volume (43%) compared to the moister site (47%; Table 3), an apparently modest difference but statistically highly significant [*F*_(1,__6)_ = 23.87, *P* = 0.003]. Differences among populations were also significant [*F*_(18,__468)_ = 1.87, *P* = 0.016], with average percentages of bark volume ranging from 42 to 47% (Supplementary Table S2). Site by population interaction was not significant [*F*_(18,__468)_ = 0.88, *P* = 0.608; Supplementary Table S3], indicating a similar population ranking for percentage of bark between sites. Total volume as a covariate for percentage of bark was highly significant [total volume: *F*_(1,__430)_ = 112.98, *P* < 0.001], as well as volume × site interaction [*F*_(1,__430)_ = 7.61, *P* = 0.006]. The rest of factors and interactions were not significant (Supplementary Table S4). The allometric effect of total volume on percentage of bark volume was negative (Figure 3). While at lower total volumes, this allometric effect was similar between sites, at higher total volumes differences between sites were magnified.

**Table 3:** Minimum, mean and maximum percentage of bark volume (%V_B_) with confidence intervals (CI) for each test site (*n* = 512 trees).

Site	min. %V_B_ [CI]	Mean %V_B_ [CI]	max. %V_B_ [CI]
Moister	43.7 [39.0–48.4]	46.6 [45.3–47.9]	48.5 [45.6–51.5]
Drier	39.4 [35.5–43.4]	42.3 [41.6–44.2]	47.1 [43.7–50.4]

Regarding the allometric (SMA) analysis, half of the populations showed constant investment in bark with tree size, i.e., isometric allometric exponents (*b* = 1), at the drier site and the other half, decreasing investment with tree size (*b* < 1). At the moister site, most populations had isometric allometric exponent (*b* = 1), except three populations showing decreasing investment in bark (*b* < 1; Supplementary Table S5). In general, populations at the moister site showed slopes closer to 1 (mean *b* = 0.92) than the same populations at the drier site (mean *b* = 0.86; differences between slopes were significant: LRT = 5.56, *df* = 1, *P* = 0.018).

### Plastic and Genetic Effects on Bark Thickness at Breast and Basal Height

Absolute bark thickness was significantly lower (*P* < 0.001) at the site with lower water availability both at breast and basal heights (Table 4 and Supplementary Table S6). We performed this analysis for all 19 populations taken together and specifically for the 13 Iberian populations (for which we have fire record data). Results for the Iberian populations followed the same pattern as those for all populations, so we only show the results for all populations.

**Table 4:** Minimum, mean and maximum absolute bark thickness at basal (BT10) and breast heights (BT130) with confidence intervals (CI) for each test site (*n* = 512 trees).

Site	BT10 (mm)	CI (mm)	BT130 (mm)	CI (mm)
Moister	23.5	22.8–24.2	12.5	11.8–13.1
Drier	20.6	20.1–21.2	8.1	7.6–8.5

**FIGURE 4:**
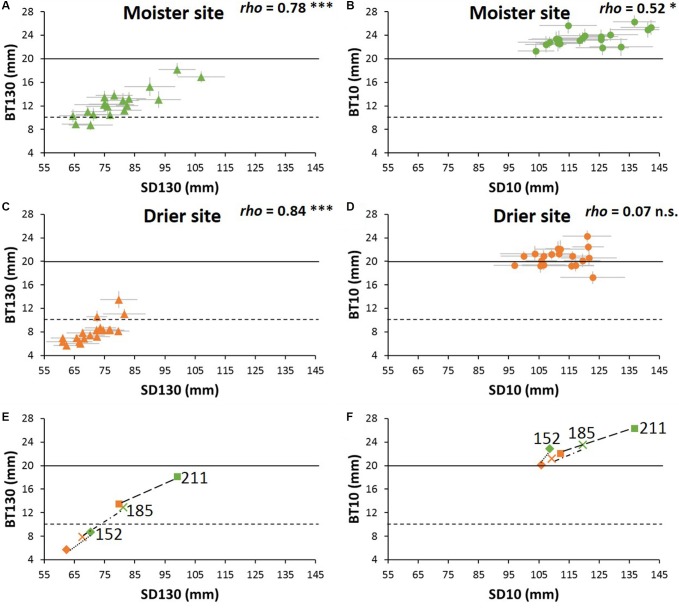
**(A–D)** Bark thickness vs. sapwood diameter of *P. halepensis* populations at each experimental site. BT130 and BT10 are bark thickness at breast height and at the tree base, respectively, SD130 and SD10 are sapwood diameter at breast height and at the tree base, respectively. Horizontal black lines represent the assumed values of critical bark thickness for cambium survival (solid line: 10 mm; dashed-line: 20 mm). Significance for Spearman correlations between bark thickness and sapwood diameter at: ^∗∗∗^*P* < 0.001, ^∗∗^*P* < 0.01, ^∗^*P* < 0.05, n.s. = no significant. **(E,F)** Variation between sites of the relationship between critical bark thickness and sapwood diameter of three representative *P. halepensis* populations. Numbers are the population code as in Supplementary Table S1. Green symbols indicate moister site and orange symbols drier site (*n* = 512 trees).

At the tree base, all populations showed bark thicker than the low critical threshold (10 mm) at both experimental sites (Figure 4A–D). By contrast, while all populations reached the high threshold thickness of 20 mm at the moister site (21.3–26.3 mm, Supplementary Table S7), not all of them did at the drier site (17.3–24.3 mm, Supplementary Table S7 and Figure 4). As for the bark thickness at breast height, only three populations exceed the 10 mm thickness threshold at the drier site. At the site with higher water availability, most of the populations showed a bark thickness higher than 10 mm, but below 20 mm- only two populations did not reach the low critical value of 10 mm-. At breast height, the range of differences among populations within sites was more than twofold. Moreover, the ranking of populations for bark thickness at breast height remained rather stable: populations with higher growth rates (from Greece and Italy) had higher BT130, while the populations with lower growth rates (Tunisia and southern Spain) showed lower BT130 at both test sites. However, at the tree base the populations ranking varied considerably between the drier and moister sites, without clear patterns. Only one of the faster-growing populations (P211) had high BT10 at both test sites and one population from southern Spain remained below the average at both sites (population P172, Supplementary Table S7).

Bark thickness at breast height was closely linked to sapwood diameter among populations, whereas at the tree base bark thickness was little correlated to sapwood diameter, especially at the drier site (Figure 4A–D). To help understanding the differences in plasticity of bark thickness between sites, we depicted the sapwood-bark trends between sites for three extreme provenances: one that did not reach the minimum critical bark thickness even at the moister site (P152), another one in which the plasticity conducted to a different critical bark thickness between sites (P185), and finally a third population that achieved the critical bark thickness even at the drier site (P211, Figure 4E,F).

### Relationship of Bark Thickness With Seed Source Environment

**FIGURE 5:**
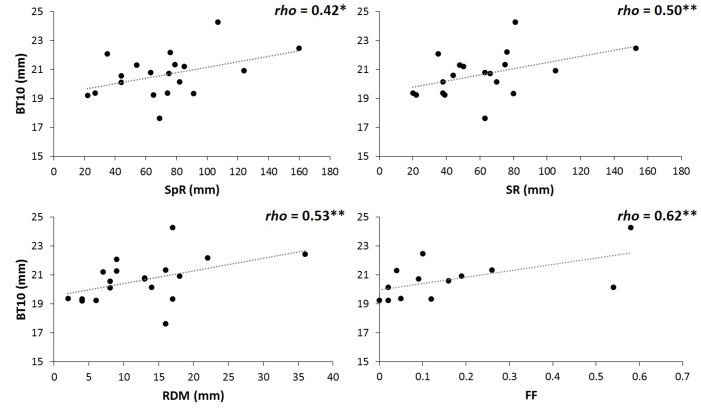
Spearman correlations between basal bark thickness at the drier site (BT10) and spring rainfall (SpR), summer rainfall (SR), rainfall of the driest month (RDM), and fire frequency (FF). Significance for Spearman correlations at: ^∗∗∗^*P* < 0.001, ^∗∗^*P* < 0.01, ^∗^*P* < 0.05, n.s. = no significant. (*n* = 19 populations for correlations with environmental variables; *n* = 13 Iberian populations for correlations with fire frequency).

Parallel analysis associated with PCA for environmental variables at the populations’ origins revealed two principal components (variance explained: PC1 = 45%, PC2 = 36%; Martín-Sanz, 2018). Considering loadings above 0.80, the first principal component was positively related to spring and summer rainfalls, as well as to the precipitation of the driest month. PC2 was negatively related to the continentally index and positively to autumn rainfall (see Supplementary Table S8). Pooling together data of both sites, BT10 was correlated (at 90% confidence, *P* = 0.10) with summer rainfall and precipitation of the driest month (Spearman rho = 0.46, *P* = 0.049, rho = 0.42, *P* = 0.072, respectively). BT10 just at the drier site was correlated with summer and spring rainfall, and precipitation of the driest month (rho = 0.50, *P* = 0.031, rho = 0.42, *P* = 0.074, and rho = 0.53, *P* = 0.020, respectively, Figure 5). At the moister site we did not find any association between bark thickness and environmental variables (Supplementary Table S9). Natural fire frequency at the original populations’ habitat was positively correlated with bark thickness at the tree base, but only at the drier site (Spearman rho = 0.62, *P* = 0.024).

## Discussion

### Allometric, Plastic, and Genetic Effects on Bark Allocation

This study highlights the existence of phenotypic plasticity for bark traits in a conifer, responding to different resource availability among environments. Using a replicated common garden experiment at two highly contrasting sites we could differentiate environmental, allometric and genetic effects (Merilä and Hendry, 2014).

Following our expectations, at the drier site our sampled *P. halepensis* trees allocated fewer resources to the bark, even considering the large difference in size among sites (see Figure 3 where the percentage of bark volume is compared between tree equal sizes -equal total volumes-). Noteworthy, the decreasing relationship between the percentage of bark volume and the total volume was more pronounced at the drier site, confirmed also by the allometric analyses.

Instead of using the usual ratio with stem diameter (see for example, Paine et al., 2010; Pausas, 2015; Midgley and Lawes, 2016) we followed a new methodological approach intending to be closer to the real allometry between the bark and the rest of the tree. We considered this better defined by comparing the percentage of bark volume vs. the total volume of the trunk, considering also that all stem was sapwood in our sampled *P. halepensis* trees (Supplementary Figure A.1). As expected, due to the well-documented relationship between bark thickness and stem diameter (Adams and Jackson, 1995; Pinard and Huffman, 1997; Lawes et al., 2013; Poorter et al., 2014), bark volume was closely related to total volume. This allometric effect, which has an adaptive significance, implies that the evolutionary forces that act on plant size will produce changes in bark thickness (Rosell, 2016; Rosell et al., 2017). Due to this relationship, when the goal is to compare differential allocation to the bark among species or populations, plant size must be taken into account (Hempson et al., 2014; Poorter et al., 2014; Rosell and Olson, 2014). However, while differential relative allocation to bark is essential under a functional-evolutionary perspective, survival to fire clearly depends on absolute bark thickness (Midgley et al., 2010; Lawes et al., 2011b). Confirming our main hypothesis, the lower allocation to bark in the growth-limiting environment led to lower absolute bark thickness bark, such that at 18 years of age (close to an average-low fire return interval for *P. halepensis*) trees did not achieve the critical thickness of 20 mm. Moreover, our results confirmed a strong population genetic effect on absolute bark thickness, with eastern Europe populations showing thicker bark, and populations from North Africa and Southern Spain displaying thinner bark. Difference of bark thickness at breast height among extreme populations was more than twofold (7.2 mm -population 152– and 15.8 mm –population 211–). This difference among populations is comparable to that found for trees of similar height in *P. pinaster* (Tapias et al., 2004), one of the pine species with best known strong ecotypic variation (see for example Alía et al., 1995). Moreover, plasticity of bark absolute thickness was rather homogeneous among provenances, hence the site-related scarcity of resources led to lower bark thickness affecting all populations alike.

While it has been shown that inner living bark is involved in the storage and translocation of water and photosynthates (Srivastava, 1964; Scholz et al., 2007; Romero, 2014), thermal protection depends on the outer dead bark thickness (Romero and Bolker, 2008; Pásztory and Ronyecz, 2013), rather than on the total bark thickness (Graves et al., 2014). Moreover, it seems that the decreasing rate in outer bark thickness with tree height is greater in fire-resistant species than in fire-sensitive ones, in contrast to inner bark thickness which diminishes with tree height or total bark thickness which is maintained along the bole (Graves et al., 2014). Therefore, differentiating between inner and outer bark thicknesses in future studies will help us to better understand the ecology and functional role of bark.

### Environmental Correlations and Life-History Implications

The environmental correlations found in this work suggest the existence of ecotypic patterns in this species that deserve further investigation to understand their evolutionary meaning. We found a highly expected significant correlation between higher frequency of natural forest fires and thicker basal bark. Among the environmental characteristics at the provenances’ seed source, the most significant correlation occurred between higher rainfall and thicker bark. These results combined may appear counter-intuitive, but it is necessary to bear in mind that in Mediterranean ecosystems higher rainfall leads to high fuel accumulation that may increase fire hazard during yearly summer droughts. Actually, permanently dry forests are usually less likely to burn due to lower fuel loads, even when trees may be physically more flammable (Fuhlendorf and Smeins, 1997; Kitzberber et al., 1997; Fernandes, 2013). A more accurate estimation of the fire regime including not only fire frequency data but also intensity, spread patterns or seasonality, and amount and type of fuel, would indeed allow us to better understand the role of fire on the variation of bark thickness in *P. halepensis*.

As an obligate seeder, the vital strategy of *P. halepensis* is the maintenance of an aerial seed bank in serotinous cones, enough for the persistence of the population in case of fire (Tapias et al., 2001; Ne’eman et al., 2004). Therefore, it is essential that trees can survive low or moderately intense fires until accumulating this “sufficient” aerial seed bank. This is usually achieved when *P. halepensis* trees are between 15 and 20 years old (Moreira et al., 2011), but both intraspecific variation and plasticity can largely modify that reference age (Martín-Sanz et al., 2016). Lacking specific data on the critical minimum bark thickness for this species, we chose two extreme values based on the literature (see van Mantgem and Schwartz, 2003; Wesolowski et al., 2014; Pausas, 2015; Madrigal et al., 2019). We found that our trees generally reached the critical bark thickness at the base, but not at breast height. This is consistent with a greater thermal insulation at the stem base and a steep bark tapering along the bole found in several Mediterranean pine species (De Ronde, 1982; Pageaud, 1991; Jackson et al., 1999). This can be regarded as an adaptive solution to reduce immaturity risk (Lamont et al., 1991; Keeley et al., 1999), i.e., ensuring individual survival until reaching a sufficient aerial seed bank enabling recruitment after lethal fires. Importantly, our studied trees did not reach the critical breast height bark thickness of 20 mm and, at the base of the tree, this value was only achieved at the moister site. Therefore, as predicted, the plasticity associated to different resource availability (mostly water availability during the vegetative period, since light, nutrients and CO_2_ are not limiting in our study sites) clearly affects the probability of *P. halepensis* to survive fires; the lesser resources, the lower expected survival facing fires. Interestingly, depending on the species and habitats, variable and even opposite relationships have been found between bark thickness and productivity or soil fertility (Climent et al., 2004; Schubert, 2014; Jager et al., 2015; Richardson et al., 2015). However, as far as we know, our study is the first to assess the strict environmental effect separated from the genotype-environment interaction; thus, the comparison with earlier works is not straightforward.

The maximum life expectancy of *P. halepensis* due to fire return interval is usually estimated between 20 and 50 years, while the average is about 25 years (Agee, 1998; Vázquez de la Cueva and Moreno, 1998; Arianoutsou, 2001; Tessler, 2012). However, fire interval could be as short as 6 years in some areas of its natural distribution (Tessler, 2012). When fire interval is shorter than 15 years, *P. halepensis* recruitment would be limited, and its populations may totally disappear (Roitemberg and Ne’eman, 2000; Eugenio et al., 2006; Herman, 2009; Tessler et al., 2014). Therefore, the age of our sampled trees -18 years- is highly meaningful considering the species’ life-history.

Preceding studies in *P. halepensis* have shown relevant ecotypic trends for growth, reproduction and water use efficiency such that provenances from dry continental environments showed early and intense reproduction and serotiny, as well as conservative water use in detriment of vegetative growth (Ferrio et al., 2003; Voltas et al., 2008, 2015; Santos-del-Blanco et al., 2013). Therefore, adaptive geographic variation of this species seems to be derived from differential resource allocation among key life-history processes (growth, reproduction, fire tolerance, constitutive and induced defenses, and water stress tolerance; Sampedro et al., 2014; Climent et al., 2015).

## Conclusion

In addition to the expected allometric effect on bark thickness, so that bigger trees had thicker bark, we found phenotypic plasticity for different bark traits in *Pinus halepensis*. Our results confirmed that growth-limiting environments hampered bark thickness such that trees did not achieve the critical bark thickness necessary to survive fires at 18 years of age, a time close to an average-low fire return interval for this species, while the relative allocation to reproduction is maximized under growth-limiting conditions (Santos-del-Blanco et al., 2013; Martín-Sanz et al., 2016). This happened not only because of lower growth, but also due to a higher negative allometry of bark thickness relative to stem size at the site with lower water availability. This could be detrimental for the resilience of this species’ populations under the more intense droughts and more frequent and severe forest fires driven by ongoing climate change. Moreover, despite *P. halepensis* is considered typically an obligate seeder, our study revealed that some populations can achieve a sufficient bark thickness to survive surface and moderately-intense fires. This suggests a more variable adaptive strategy to cope with fire than has been considered so far for this species (see Ducrey et al., 1996; Rigolot, 2004; Fernandes et al., 2008); the correlations found between bark traits, fire regime and local climate among populations support the ecotypic nature of this intraspecific variation. To our knowledge, this is the first study providing experimental evidence of plasticity for this key adaptive trait in interaction with population differentiation. We still lack direct experimental data on the critical bark thickness for *P. halepensis* survival, and on the possible differences in bark morphology, its internal structure and the rate of bark thickness tapering along the entire bole; aspects that can be critical for tree survival. Moreover, the possible trade-offs with other key processes (namely reproduction or defense) deserve further investigation under a Climate Change scenario.

## Author Contributions

RM-S and JC conceived and designed the study. RM-S carried out fieldwork. AV collected and prepared fire data. RM-S and RS-M designed and performed the statistical analysis. RM-S wrote the original draft. JC and HP revised the manuscript. All authors have read and approved the final manuscript.

## Conflict of Interest Statement

The authors declare that the research was conducted in the absence of any commercial or financial relationships that could be construed as a potential conflict of interest.
