# Supplementary material for: How Does Water Availability Affect the Allocation to Bark in a Mediterranean Conifer?
Source: Front Plant Sci. 2019 May 21;10:607. doi: 10.3389/fpls.2019.00607 (PMC6536605; doi:10.3389/fpls.2019.00607)
Supplement: Supplementary file 1 [file Data_Sheet_1.PDF]

Supplementary Material

How Does Environment Affect the Allocation to Bark in a Mediterranean Conifer?

Ruth C. Martín-Sanz*, Roberto San-Martín, Hendrik Poorter, Antonio Vázquez and José Climent

***Correspondence:** Ruth C. Martín Sanz: ruth.martin.sanz@gmail.com

frontiers

Table S1. Geographic and climatic information about the 19 native *Pinus halepensis* populations used in this study.

Country	Code	Region	Location	Longitude	Latitude	Altitude (m)	T (⁰C)	R (mm)	SR (mm)
Spain	11	Cataluña	Cabanellas	2º47'00"E	42º14'51"N	221	14.2	858	208
Spain	21	Cataluña	Tivissa	0º50'06"E	42º19'46"N	368	15.3	566	75
Spain	92	Maestrazgo - Los Serranos	Tuéjar	1º09'33"W	39º49'06"N	665	15	385	91
Spain	111	Maestrazgo - Los Serranos	Benicasim	0º01'33"E	40º04'37"N	449	14.2	699	90
Spain	101	Levante Interior	Tibi	0º38'55"W	38º31'08"N	993	13	503	58
Spain	105	Levante Interior	Bicorp	0º51'30"W	39º06'12"N	619	14.75	598.5	80.5
Spain	131	Levante Interior	Villajoyosa	0º18'14"W	38º29'44"N	98	16.8	431	51
Spain	142	Bética Septentrional	Monovar	0º57'27"W	38º23'05"N	760	14.7	387	51
Spain	152	Bética Meridional	Benamaurel	2º44'19"W	37º42'05"N	914	14.3	394	44
Spain	154	Bética Meridional	Santiago de la Espada	2º28'03"W	38º13'35"N	761	13.6	608	66
Spain	157	Bética Meridional	Alhama de Murcia	3º01'19"W	37º45'10"N	818	15.6	433	29
Spain	172	South	Carratraca	4º50'01"W	36º50'32"N	643	14.85	699.5	33
Spain	173	South	Frigiliana	3º55'13"W	36º49'03"N	583	15.2	696	26
Spain	182	Mallorca	Palma de Mallorca	2º56'26"E	39º08'56"N	46	16.7	563	40
Spain	185	Mallorca	Son Marti/Calviá	2º29'13"E	39º35'14"N	272	15.8	526	55
Greece	211	Greece	Istaia-eyboia	23º30'46"E	38º44'29"N	27	17.47	506	35
Greece	214	Greece	Kassandra	23º54'27"E	40º05'13"N	173	14.37	510	61
Italy	231	Italy	Litorale Tarantino	17º07'04"E	40º37'08"N	106	15.17	551	76
Tunisia	241	Tunisia	Thala	8º39'00"E	35º33'60"N	527	14.89	467	63

Notes: Code: population code; T: mean annual temperature; R: annual rainfall; SR: summer rainfall (June, July and August). All previous climatic variables from WORLDCLIM v.1.4 (Hijmans et al., 2005). The 13 Iberian populations are in black font and the non-Iberian populations are in blue font.

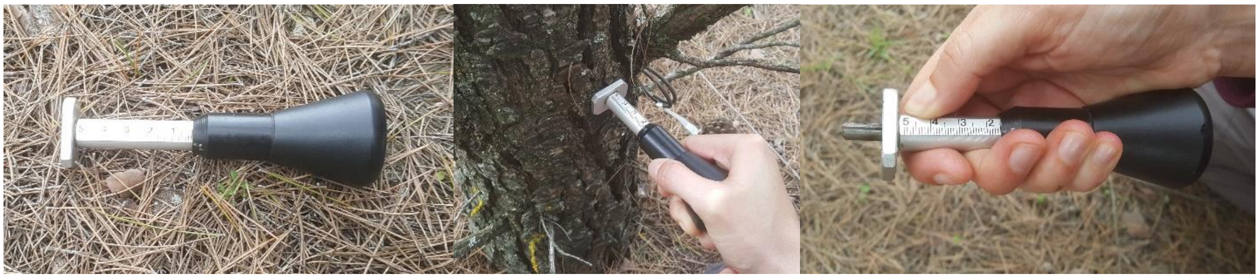

Figure S1. (A) Photograph of a standard bark gauge, (B) measuring bark thickness with the bark gauge and (C) standard bark gauge indicating the thickness of the measured bark.

Appendix A. Absence of heartwood in the sampled individuals

Background

Heartwood formation in woody plants is a consequence of the regulation of conductive xylem adequate to the balance between water acquisition and transpiration (see for example Moreno et al., 2013). Heartwood formation follows the irreversible embolism of the water conducting elements at inner sapwood rings with the death of living parenchyma cells and deposition of extractives with protective properties (Bamber, 1976; Spicer, 2005). The onset of heartwood formation is highly variable among species and environments, with strong allometric effects combined with tree age (Yang and Hazenberg, 1991; Climent et al., 2003; Mäkelä and Valentine, 2006). Conifers in general show a particularly delayed heartwood formation compared to broadleaves due to their particular xylem structure (Hillis, 2012). In pines, heartwood can appear typically between 20 and 30 years, with early growth rates affecting significantly (and positively) its precocity and early size (Wilkes, 1991; Arencibia and Climent, 2009). Moreover, heartwood starts earlier at a certain height within the stem (1 to 4 m) progressing both towards the tree base and upwards in the stem (Climent et al., 2003; Stokes and Berthier, 2000). Generally, warm-dry environments accelerate heartwood formation compared to cold-wet environments (Climent et al., 2002; Moreno et al., 2013).

Sampling and results

Wood cores were extracted at an intermediate height between tree base and breast height (60 cm) from a subsample of 3 trees of all 19 provenances at each site, randomly chosen. Cores were photographed immediately after extraction (**Figure A.1.**) to detect non-functional sapwood (easily identified in fresh samples) and eventually heartwood. As expected, we found no heartwood in any wood core. Symptoms of partial loss of conductivity in inner wood rings was identified in less than 5 % of the samples. This would lead to the onset of heartwood in a few years depending on water availability.

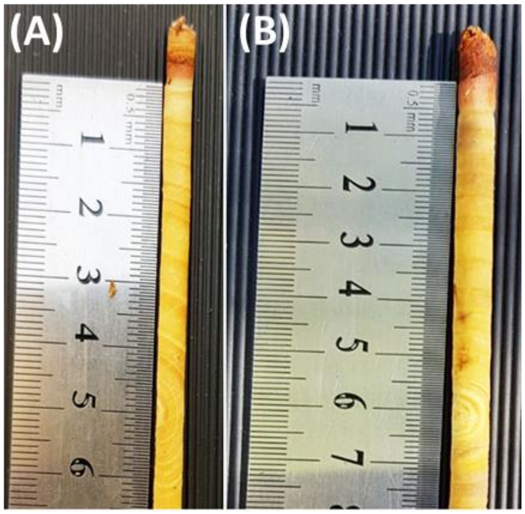

Figure A.1. Photograph of two wood cores extracted from a subsample of trees at both sites of the common garden. A) Fully functional sapwood. B) Slightly drier sapwood at inner rings (<5% of samples).

References

Arencibia, L. F., and Climent, J. M. (2009). Desarrollo de la madera de duramen (tea) en las masas artificiales de pino canario en Gran Canaria. *Montes*, 99: 15–20.

Bamber, R. K. (19769. Heartwood, its function and formation. *Wood Science and Technology,* 10, 1–8.

Climent, J. M., Chambel, M. R., Pérez, E., Gil, L., and Pardos, J. (2002). Relationship between heartwood radius and early radial growth, tree age, and climate in *Pinus canariensis*. *Can. J. For. Res.,* 32, 103–111.

Climent, J. M., Chambel, M.R., Gil, L., and Pardos, J.A. (2003). Vertical heartwood variation patterns and prediction of heartwood volume in *Pinus canariensis* Sm. *For. Eco. Mang.,* 174, 203–211.

Mäkelä, A., and Valentine, H.T. (2006). Crown ratio influences allometric scaling in trees. *Ecology*, 87, 2967–2972.

Moreno, C. J., Raymond, C. A., and Walker, J. C. F. (2013). Development of heartwood in response to water stress for radiata pine in Southern New South Wales, Australia. *Trees Struct.-Funct.,* 27; 607–617.

Spicer, R. (2005). Senescence in secondary xylem: heartwood formation as an active developmental program. In: *Vascular transport in plants* (pp. 457-475). Academic Press.

Stokes, A., and Berthier, S. (2000). Irregular heartwood formation in *Pinus pinaster* Ait. is related to eccentric, radial, stem growth. *For. Eco. Mang.,* 135, 115–121.

Wilkes, J. (1991). Heartwood development and its relationship to growth in *Pinus radiata*. *Wood Science and Technology*, 25, 85–90.

Yang, K., and Hazenberg, G. (1991). Sapwood and heartwood width relationship to tree age in *Pinus banksiana*. *Can. J. For. Res.,* 21, 521–525.

Table S2. Mean percentage of bark volume (%V_B) and confidence intervals for each population obtained through general linear mixed models.

**Code**	**%V_B [CI]**
11	43.5 [41.8-45.2]
21	45.9 [43.9-47.9]
92	46.0 [43.2-48.8]
101	41.9 [39.2-44.6]
105	44.2 [41.7-46.7]
111	46.3 [44.2-48.3]
131	45.2 [43.3-47.1]
142	43.0 [40.8-45.2]
152	45.0 [42.7-47.4]
154	43.2 [41.2-45.2]
157	45.2 [43.5-46.8]
172	43.8 [42.0-45.7]
173	43.9 [42.0-45.8]
182	46.4 [44.2-48.6]
185	45.6 [43.8-47.5]
211	47.4 [44.9-49.9]
214	42.2 [39.4-44.9]
231	44.8 [42.0-47.6]
241	46.7 [44.4-49.1]

Notes: Code is population code (see **Table S1**). The 13 Iberian populations are in black font and the non-Iberian populations are in blue font.

Table S3. Type III test of fixed effects table for the general linear model done to analyze the percentage of bark volume by site and population.

	Num DF	Den DF	F-Value	Pr > F
**Site**	1	6	23.87	0.0028
**Population**	18	468	1.87	0.0162
**Site x Population**	18	468	0.88	0.6082

Num DF: the degrees of freedom of the numerator; Den DF: the degrees of freedom of the denominator.

Table S4. Type III test of fixed effects table for the general linear model done to analyze the percentage of bark volume by site and population with total volume as a covariate.

	Num DF	Den DF	F-Value	Pr > F
**Site**	1	6	2.19	0.1898
**Population**	18	430	1.35	0.1520
**Site x Population**	18	430	1.42	0.1154
**Total Volume (V_T)**	1	430	112.98	<.0001
**V_T x Site**	1	430	7.61	0.0060
**V_T x Population**	18	430	1.11	0.3428
**V_T x Site x Population**	18	430	1.50	0.0865

Num DF: the degrees of freedom of the numerator; Den DF: the degrees of freedom of the denominator.

Table S5. Bark allometric exponents (b) for bark volume of each population at the two experimental sites (confidence intervals in brackets). Significant *P* value indicates that b is different from 1 (isometric coefficient). r^2 and significance for the standardized major axis regression (SMA).

	**Allometric exponent (α)**		***P* value**		**r^2**	
**Code**	**Moister site**	**Drier site**	**Moister site**	**Drier site**	**Moister site**	**Drier site**
11	0.87 [0.71-1.08]	0.89 [0.70-1.12]	0.184	0.280	0.82***	0.87***
21	0.80 [0.68-0.94]	0.99 [0.78-1.25]	**0.011**	0.896	0.97***	0.86***
92	0.93 [0.85-1.02]	0.77 [0.40-1.49]	0.098	0.410	0.99***	0.82***
101	0.85 [0.66-1.10]	0.97 [0.77-1.22]	0.201	0.795	0.79***	0.91***
105	0.98 [0.83-1.16]	0.97 [0.61-1.56]	0.808	0.900	0.92***	0.45**
111	0.87 [0.74-1.01]	0.86 [0.75-0.98]	0.068	**0.027**	0.93***	0.94***
131	0.90 [0.78-1.04]	0.95 [0.76-1.17]	0.131	0.577	0.94***	0.94***
142	0.94 [0.84-1.04]	0.87 [0.72-1.06]	0.190	0.144	0.98***	0.84***
152	0.82 [0.76-0.89]	0.75 [0.61-0.92]	**<0.001**	**0.009**	0.98***	0.88***
154	0.98 [0.86-1.11]	0.79 [0.63-0.98]	0.725	**0.038**	0.97***	0.93***
157	0.92 [0.82-1.03]	0.73 [0.56-0.97]	0.147	**0.034**	0.98***	0.84***
172	0.98 [0.88-1.10]	0.86 [0.58-1.27]	0.761	0.422	0.97***	0.81***
173	1.02 [0.92-1.13]	0.72 [0.55-0.95]	0.711	**0.025**	0.97***	0.91***
182	0.84 [0.75-0.95]	0.78 [0.69-0.87]	**0.008**	**0.001**	0.98***	0.95***
185	0.96 [0.83-1.12]	0.82 [0.72-0.94]	0.613	**0.009**	0.97***	0.98***
211	0.83 [0.61-1.13]	1.01 [0.85-1.20]	0.212	0.881	0.82***	0.93***
214	0.74 [0.53-1.03]	0.77 [0.59-1.00]	0.073	**0.050**	0.88***	0.88***
231	0.98 [0.83-1.16]	1.42 [1.00-2.04]	0.802	0.052	0.86***	0.70**
241	0.92 [0.76-1.12]	0.79 [0.67-0.94]	0.396	**0.012**	0.90***	0.95***

Notes: Code is population code (see **Table S1**). The 13 Iberian populations are in black font and the non-Iberian populations are in blue font. Significant *P* values in bold case ($P < 0.001$). *** < 0.001, ** < 0.01, * < 0.05.

Table S6. Type III test of fixed effects table for the general linear model done to analyze the absolute bark thickness by site and population at breast (A) and basal heights (B).

(A) Breast height	Num DF	Den DF	F-Value	Pr > F
**Site**	1	6	171.02	<.0001
**Population**	18	468	10.55	<.0001
**Site x Population**	18	468	0.99	0.4726
**(B) Basal height**				
**Site**	1	6	57.68	0.0003
**Population**	18	468	2.07	0.0062
**Site x Population**	18	468	1.37	0.1432

Num DF: the degrees of freedom of the numerator; Den DF: the degrees of freedom of the denominator.

Table S7. Mean bark thickness and confidence intervals at breast height (BT130) and at the tree base (BT10) for each population and site obtained through general linear mixed models.

	**BT130 [CI] (mm)**		**BT10 [CI] (mm)**	
**Code**	**Moister site**	**Drier site**	**Moister site**	**Drier site**
11	12.2 [11.1-13.2]	8.2 [7.3-9.0]	24.6 [23.1-26.2]	22.5 [21.0-23.9]
21	10.5 [9.0-12.0]	6.9 [6.1-7.6]	25.6 [23.0-28.2]	20.9 [18.9-22.9]
92	11.9 [9.2-14.7]	6.0 [4.9-7.1]	23.3 [20.5-26.2]	21.3 [18.8-23.8]
101	11.3 [9.5-13.0]	7.5 [6.3-8.6]	21.9 [19.4-24.3]	19.3 [17.3-21.4]
105	11.0 [9.2-12.9]	8.4 [7.2-9.6]	22.5 [19.8-25.2]	20.1 [17.9-22.3]
111	13.8 [11.9-15.6]	8.3 [6.9-9.7]	24.1 [22.1-26.2]	24.3 [22.4-26.2]
131	13.5 [11.3-15.6]	7.0 [5.9-8.1]	22.6 [20.8-24.4]	19.3 [16.9-21.6]
142	13.2 [10.2-16.1]	8.3 [7.4-9.2]	25.5 [23.0-28.1]	20.9 [19.4-22.4]
152	8.7 [7.1-10.4]	5.7 [5.0-6.4]	22.9 [21.0-24.7]	20.1 [19.0-21.2]
154	13.2 [11.0-15.4]	8.3 [7.0-9.7]	23.3 [20.8-25.7]	20.6 [18.2-23.0]
157	10.5 [8.4-12.7]	7.2 [6.4-7.9]	23.4 [21.9-25.9]	21.3 [19.7-22.9]
172	8.9 [7.5-10.2]	6.4 [5.2-7.6]	21.4 [19.1-23.6]	20.0 [18.8-21.2]
173	12.3 [9.7-15.0]	8.7 [7.3-10.1]	23.2 [20.9-25.4]	19.2 [17.9-20.6]
182	12.2 [10.0-14.5]	7.0 [5.9-8.1]	23.9 [21.6-26.2]	19.4 [18.1-20.6]
185	12.9 [10.6-15.3]	7.9 [6.5-9.2]	23.6 [21.6-25.6]	21.2 [19.6-22.8]
211	18.2 [16.1-20.3]	13.5 [10.8-16.2]	26.3 [24.2-28.4]	22.1 [19.2-25.0]
214	16.9 [15.1-18.8]	11.1 [9.5-12.7]	25.3 [22.8-27.8]	17.3 [15.2-19.3]
231	15.3 [12.2-18.4]	10.6 [8.6-12.6]	22.0 [19.0-25.0]	22.2 [19.7-24.6]
241	10.3 [8.1-12.5]	6.3 [5.5-7.1]	22.6 [20.0-25.3]	20.9 [19.5-22.4]

Notes: Code is population code (see **Table S1**). The 13 Iberian populations are in black font and the non-Iberian populations are in blue font.

Table S8. Results from Principal Component Analysis used to reduce the number of environmental variables obtained from WorldClim v1.4 for 19 *Pinus halepensis* source populations. Environmental variables with loadings > |0.80| were selected for correlation analysis and are in bold case.

Variable	PC1	PC2
**Summer rainfall**	**0.95**	-0.16
**Spring rainfall**	**0.94**	0.05
**Autumn rainfall**	0.09	0.82
**Rainfall of driest month**	**0.90**	-0.21
Annual rainfall	0.61	0.51
Mean temperature of warmest month	-0.79	-0.30
**Mean temperature of coldest month**	-0.15	**0.94**
**Temperature annual range***	-0.43	**-0.82**
Annual mean temperature	-0.47	0.75
Importance of components		
Proportion of variance explained	0.45	0.36
Cumulative proportion of variance	0.45	0.80

*Difference between maximum temperature of the warmest month and minimum temperature of the coldest month.

Table S9. Correlations between mean bark thickness values (at breast height: BT130 and at tree base: BT10) from *Pinus halepensis* trees grown in a common garden experiment replicated in two contrasting sites and the six selected environmental variables representing average conditions in source populations. Significant correlations (< 0.10) are indicated in bold.

Environmental Variable	Moister site		Drier site		2 sites	
	**BT130**	**BT10**	**BT130**	**BT10**	**BT130**	**BT10**
Summer rainfall	0.03	0.11	0.08	**0.50**	0.07	**0.42**
Spring rainfall	0.02	0.16	-0.01	**0.42**	0.05	0.37
Autumn rainfall	0.23	0.26	0.26	-0.12	0.24	0.01
Rainfall of driest month	0.12	0.16	0.24	**0.53**	0.17	**0.46**
Mean temperature of coldest month	0.20	0.13	0.07	0.15	0.23	0.13
Temperature annual range*	-0.20	-0.14	-0.00	-0.15	-0.15	-0.21
Fire frequency	0.15	0.14	0.19	**0.62**	0.16	0.37

*Difference between maximum temperature of the warmest month and minimum temperature of the coldest month.
